# Structure of *Helicobacter pylori* dihydroneopterin aldolase suggests a fragment-based strategy for isozyme-specific inhibitor design

**DOI:** 10.1016/j.crstbi.2023.100095

**Published:** 2023-01-30

**Authors:** Gary X. Shaw, Lixin Fan, Scott Cherry, Genbin Shi, Joseph E. Tropea, Xinhua Ji

**Affiliations:** a Center for Structural Biology, National Cancer Institute, National Institutes of Health, 1050 Boyles Street, Frederick, MD, 21702, USA; b Basic Research Program, Frederick National Laboratory for Cancer Research, Small-angle X-ray Scattering Core Facility, National Cancer Institute, National Institutes of Health, 1050 Boyles Street, Frederick, MD, 21702, USA

**Keywords:** Dihydroneopterin aldolase, Folate biosynthesis, *Helicobacter pylori*, Antibiotic, Fragment-based drug discovery, ANL, Argonne National Laboratory, APS, Advanced Photon Source, DHFS, dihydrofolate synthase, DHNA, dihydroneopterin aldolase, DHNP, 7,8-dihydroneopterin, DHPS, dihydropteroate synthase, DLS, dynamic light scattering, *D*_max_, maximum dimension, EcDHNA, *Escherichia coli* DHNA, FBDD, fragment-based drug discovery, GA, glycoaldehyde, HP, 6-hydroxymethyl-7,8-dihydropterin, HpDHNA, *Helicobacter pylori* DHNA, HPPK, 6-hydroxymethyl-7,8-dihydropterin pyrophosphokinase, ISCD, isozyme-specific contact distance, MtDHNA, *Mycobacterium tuberculosis* DHNA, MW, molecular weight, NP, neopterin, PCR, polymerase chain reaction, *P*(r), pair-distance distribution function, *R*_g_, radius of gyration, SaDHNA, *Staphylococcus aureus* DHNA, SER-CAT, Southeast Regional Collaborative Access Team, SAXS, small-angle X-ray scattering, SpDHNA, *Streptococcus pneumoniae* DHNA, TCEP, tris(2-carboxyethyl)phosphine, TEV, tobacco etch virus, wwPDB, Worldwide Protein Data Bank

## Abstract

Dihydroneopterin aldolase (DHNA) is essential for folate biosynthesis in microorganisms. Without a counterpart in mammals, DHNA is an attractive target for antimicrobial agents. *Helicobacter pylori* infection occurs in human stomach of over 50% of the world population, but first-line therapies for the infection are facing rapidly increasing resistance. Novel antibiotics are urgently needed, toward which structural information on potential targets is critical. We have determined the crystal structure of *H. pylori* DHNA (HpDHNA) in complex with a pterin molecule (HpDHNA:Pterin) at 1.49-Å resolution. The HpDHNA:Pterin complex forms a tetramer in crystal. The tetramer is also observed in solution by dynamic light scattering and confirmed by small-angle X-ray scattering. To date, all but one reported DHNA structures are octameric complexes. As the only exception, ligand-free *Mycobacterium tuberculosis* DHNA (apo-MtDHNA) forms a tetramer in crystal, but its active sites are only partially formed. In contrast, the tetrameric HpDHNA:Pterin complex has well-formed active sites. Each active site accommodates one pterin molecule, but the exit of active site is blocked by two amino acid residues exhibiting a contact distance of 5.2 ​Å. In contrast, the corresponding contact distance in *Staphylococcus aureus* DHNA (SaDHNA) is twice the size, ranging from 9.8 to 10.5 ​Å, for ligand-free enzyme, the substrate complex, the product complex, and an inhibitor complex. This large contact distance indicates that the active site of SaDHNA is wide open. We propose that this isozyme-specific contact distance (ISCD) is a characteristic feature of DHNA active site. Comparative analysis of HpDHNA and SaDHNA structures suggests a fragment-based strategy for the development of isozyme-specific inhibitors.

## Highlights

• Crystal structure of *Helicobacter pylori* DHNA in complex with pterin at 1.49 ​Å.
• *H. pylori* DHNA exists as a tetramer in crystal with well-formed active sites.
• The tetrameric *H. pylori* DHNA assembly also exits in solution.
• Structural comparison reveals an isozyme-specific feature of DHNA active sites.
• *H. pylori* DHNA structure suggests a strategy for isozyme-specific inhibitor design.

## Graphical abstract

**Figure:**
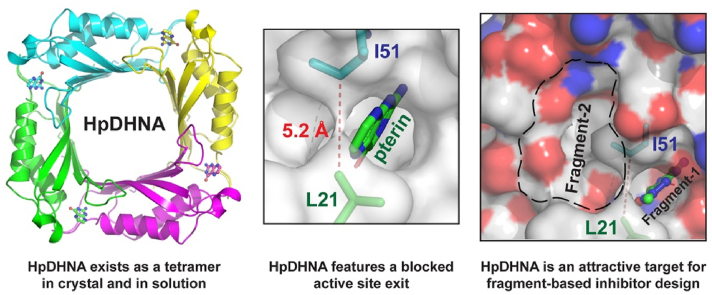

## 1 Introduction

Folate cofactors are essential for life because they are required in the biosynthesis of purine and pyrimidine bases and certain amino acids (Blakley and Benkovic, 1984). Mammals have an active transport system for deriving folates from diets, whereas most microorganisms must synthesize folates *de novo* for lack of an active transport system (Hitchings and Burchall, 1965). Therefore, the folate biosynthesis pathway is an ideal target for developing antimicrobial agents (Bermingham and Derrick, 2002; Walsh, 2003). Four mid-pathway enzymes, dihydroneopterin aldolase (DHNA), 6-hydroxymethyl-7,8-dihydropterin pyrophosphokinase (HPPK), dihydropteroate synthase (DHPS), and dihydrofolate synthase (DHFS), are particularly attractive because they are absent in mammals. Sulfonamides that inhibit DHPS were the first group of synthetic antibiotics and the clinical use of these “sulfa drugs” marks the beginning of the modern era of antimicrobial chemotherapy (Sammes, 1990; Zinner and Mayer, 2009). The combination of sulfamethoxazole (an inhibitor of DHPS) and trimethoprim (an inhibitor of dihydrofolate reductase, the last enzyme in the folate pathway) has been used to treat urinary tract, respiratory tract, and other bacterial infections (Hughes, 1997; Huovinen et al., 1995). As such, multiple targets in the folate pathway offer opportunities of developing antibiotics with synergetic effects (Zinner and Mayer, 2009).

**Fig. 1:**
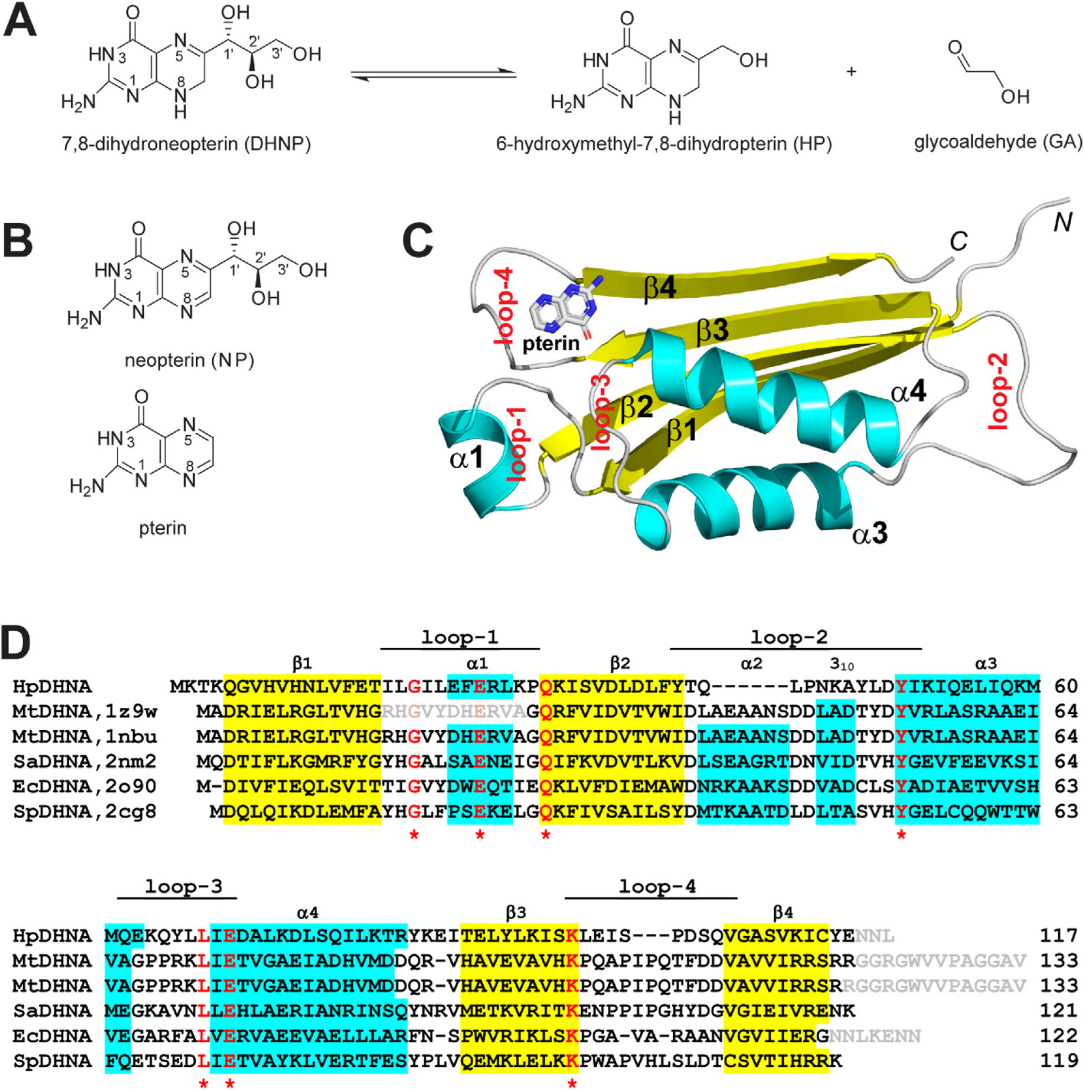
Reaction, structure, and sequence of DHNA. **(A)** DHNA-catalyzed reaction. **(B)** DHNA inhibitors neopterin (NP) and pterin. **(C)** Crystal structure of one subunit (content of the asymmetric unit) in the HpDHNA:Pterin tetramer. The protein is illustrated as a ribbon diagram (strands as arrows in yellow, helices as spirals in cyan, and loops as tubes in grey) and the ligand is shown as a stick model in atomic color scheme (N in blue, C in grey, and O in red). **(D)** Structure-based alignment of DHNA sequences. Secondary structural elements are depicted above the sequences. Strands and helices are highlighted in yellow and cyan, respectively. Strictly conserved resides are highlighted in red with a star below the sequences. Unobserved residues are indicated in grey. PDB IDs are shown in the top panel. Aligned sequences are chain A of the HpDHNA, MtDHNA, SaDHNA, and EcDHNA structures, and Chain C of the SpDHNA structure. (For interpretation of the references to color in this figure legend, the reader is referred to the Web version of this article.)

DHNA catalyzes the conversion of 7,8-dihydroneopterin (DHNP) to 6-hydroxymethyl-7,8-dihydropterin (HP, Fig. 1A) (Mathis and Brown, 1970). To date, more than 20 crystal structures have been published for DHNAs from seven bacteria, including *Staphylococcus aureus* (SaDHNA) (Blaszczyk et al., 2007; Hennig et al., 1998; Sanders et al., 2004), *Escherichia coli* (EcDHNA) (Blaszczyk et al., 2014), *Mycobacterium tuberculosis* (MtDHNA) (Goulding et al., 2005), and *Streptococcus pneumoniae* (SpDHNA) (Garcon et al., 2006), among which SpDHNA is the N-terminal component of bifunctional enzyme DHNA-HPPK. Remarkably, the trajectory of SaDHNA-catalyzed reaction has been described with crystal structures of apo-SaDHNA (PDB ID: 1DHN), the SaDHNA:DHNP complex (PDB ID: 1U68), and the SaDHNA:HP complex (PDB ID: 2DHN). This is the first structural view for DHNA-catalyzed conversion of DHNP to HP, providing basis for the functional roles of conserved amino acid residues among DHNA sequences (Hennig et al., 1998; Sanders et al., 2004). Furthermore, inhibitors have been developed for SaDHNA, among which the most potent compound (**27**), exhibiting an IC_50_ value of 68 ​nM, yields a crystal structure in complex with the enzyme at 2.7-Å resolution (Sanders et al., 2004). However, no structure has been reported for *Helicobacter pylori* DHNA (HpDHNA), which is critical for the development of *H. pylori*-specific antibiotics.

As one of the most widespread bacterial pathogens, *H. pylori* grows in the mucus coat inside human stomach of over 50% of the world population (Salama et al., 2013). Although about 90% of infected individuals are asymptomatic, *H. pylori* is responsible for considerable health risks including the development of gastric ulcers (Fallone et al., 2016). The treatment of *H. pylori* infection is difficult because of its high resistance to antibiotics, especially the first-line therapeutics clarithromycin and metronidazole (Contreras-Omana et al., 2021). A recent survey reveals the percentage of antimicrobial resistance of *H. pylori* (Alba et al., 2017), stimulating a continuous and intensive interest of developing new antibiotics to treat the infection. Among *H. pylori* species, the G27 strain has been used extensively in research (Baltrus et al., 2009). Therefore, a wealth of functional information is available for the G27 strain. Here, we report the crystal structure of *H. pylori* (strain G27) DHNA in complex with pterin (Fig. 1B) and propose a fragment-based strategy for isozyme-specific inhibitor design based on an isozyme-specific structural feature of DHNA active sites.

## 2 Materials and methods

### 2.1 Macromolecule production

The HpDHNA (strain G27) gene was amplified from genomic DNA by polymerase chain reaction (PCR) using a 10:1 mixture of PE-210 (forward PCR primer 1: GGGGACAAGTTTGTACAAAAAAGCAGGCTCGGAGAACCTGTACTTCCAG) and PPC-277 (forward PCR primer 2: GGGGACAAGTTTGTACAAAAAAGCAGGCTCGGAGAACCTGTACTTCCAG), and PPC-211 (reverse PCR primer: GGGGACCACTTTGTACAAGAAAGCTGGGTTATTAAAAGATTGTTTTCATAGCCAAATTTTCAGGC). The amplicon, coding for HpDHNA (residues M1-L117) with an N-terminal tobacco etch virus (TEV) protease recognition sequence (ENLYFQ/M1), was recombined into the cloning vector pDONR221 (Life Technologies, Carlsbad, California, USA) and sequenced. The open reading frame was moved by recombination into the destination vector pDEST527 (Protein Expression Laboratory, Frederick National Laboratory for Cancer Research, Leidos Biomedical Research Inc., Frederick, MD, USA) to produce pJT231. This plasmid directs the expression of HpDHNA with an N-terminal hexahistidine tag and an intervening TEV protease cleavage site. The fusion protein was expressed in *Escherichia coli* strain BL21-CodonPlus (DE3)-RIPL (Agilent, Santa Clara, CA, USA). Cells containing expression plasmid were grown to mid-log phase (OD_600_ of ∼0.5) at 310 ​K in LB broth (Miller's formulation) containing 100 ​μg ​ml^−1^ ampicillin, 30 ​μg ​ml^−1^ chloramphenicol, and 0.2% glucose. Overexpression was induced with 1 ​mM isopropyl β-D-1-thiogalacto-pyranoside for 4 ​h at 303 ​K. The cells were pelleted by centrifugation and stored at 193 ​K. Pilot purification trials showed poor cleavage of the fusion protein by TEV protease. To rectify this problem, sequence encoding three glycine residues were inserted into the TEV protease recognition sequence between ENLYFQ and residue M1 of HpDHNA using the QuikChange Lightning Site-Directed Mutagenesis Kit (Agilent, Santa Clara, CA, USA) with primers PPC-227 (forward mutagenic primer: GAGAACCTGTACTTCCAGGGTGGCGGTATGAAAACTAAACAAGGC) and PPC-228 (reverse mutagenic primer: GCCTTGTTTAGTTTTCATACCGCCACCCTGGAAGTACAGGTTCTC). The resulting construct, pJT250, produced fusion protein that was completely cleaved by TEV protease as indicated with an arrow in the amino acid sequence: MRSGSHHHHHHRSDITSLYKKAG SENLYFQ↓GGG-DHNA(M1-L117), in which the TEV protease recognition site is underlined.

All purification procedures were performed at 277 ​K. *E. coli* cell paste expressing the fusion protein was suspended in ice-cold buffer A (50 ​mM phosphate pH 7.5, 200 ​mM NaCl, and 25 ​mM imidazole) containing cOmplete™ EDTA-free protease inhibitor cocktail (Roche Diagnostics Corporation, Indianapolis, IN, USA). The cells were lysed with an APV-1000 homogenizer (SPX FLOW Corporation, Charlotte, NC, USA) at 69 ​MPa and centrifuged for 30 ​min at 30,000×*g*. The supernatant was filtered through a 0.45 ​μm polyethersulfone membrane and applied to a 10-ml Ni-NTA Superflow column (Qiagen Sciences, Germantown, MD, USA) equilibrated in buffer A. The column was washed to baseline with buffer A and eluted with a linear gradient of imidazole to 250 ​mM. Fractions containing recombinant fusion protein were pooled, concentrated using an Ultracel® 10 ​kDa ultrafiltration disc (EMD Millipore Corporation, Billerica, MA, USA), diluted with 50 ​mM phosphate pH 7.5, 200 ​mM NaCl buffer to reduce the imidazole concentration to about 25 ​mM, and digested overnight at 298 ​K with His_7_-tagged TEV protease (Kapust et al., 2001). The digest was applied to a 25-ml Ni-NTA Superflow column equilibrated in buffer A and recombinant protein emerged in the column effluent. The effluent was incubated overnight at 277 ​K with 10 ​mM dithiothreitol, concentrated using an Ultracel® 10 ​kDa ultrafiltration disc, and applied to a HiPrep 26/60 Sephacryl S-300 HR column (GE Healthcare Life Sciences, Piscataway, NJ, USA) equilibrated in 25 ​mM Tris pH 7.5, 150 ​mM NaCl, and 2 ​mM tris(2-carboxyethyl)phosphine (TCEP) buffer. The peak fractions containing GGG-DHNA (M1-L117) were pooled and concentrated to 30–35 ​mg ​ml^−1^ (estimated from the absorbance at 280 ​nm using a molar extinction coefficient of 10,430 ​M^−1^ ​cm^−1^ derived using the Expasy ProtPram tool) (Gasteiger et al., 2003). Aliquots were flash-frozen in liquid nitrogen and stored at 193 ​K. The final product was judged to be >95% pure by sodium dodecyl sulfate-polyacrylamide gel electrophoresis. The molecular weight was confirmed by electrospray ionization mass spectroscopy.

### 2.2 Crystallization

Crystallization of HpDHNA in the presence of HP was carried out with the Crystal Gryphon robot (Art Robbins Instruments, Sunnyvale, CA, USA) and screen kit Index (Hampton Research, Aliso Viejo, CA, USA) by sitting drop vapor diffusion at 293 ​K. The volume ratio of mixed protein solution and well solution was 1:3, i.e., 0.5 ​μl of protein sample (23.3 ​mg ​ml^−1^ HpDHNA and 2.9 ​mg ​ml^−1^ HP in 25 ​mM Tris pH 7.5, 150 ​mM NaCl, and 2 ​mM TCEP buffer) and 1.5 ​μl of reservoir solution (0.1 ​M HEPES pH 7.5 and 2.0 ​M (NH_4_)_2_SO_4_). The equilibration of each mixed drop was against 60 ​μl of the reservoir solution. A single crystal with dimensions of 0.15 ​mm ​× ​0.15 ​mm x 0.10 ​mm was transferred to a cryoprotectant containing the mother liquor with 25% (v/v) ethylene glycol and flash-cooled in liquid nitrogen.

### 2.3 Diffraction data collection and processing

X-ray diffraction data were recorded on a MARCCD225 detector at beamline 22-BM of Southeast Regional Collaborative Access Team (SER-CAT), Advanced Photon Source (APS), Argonne National Laboratory (ANL). Data were processed using the HKL-3000 program suite (Minoret al., 2006). Data collection and processing statistics are summarized in Table 1.

**Table 1:** X-ray diffraction data collection and refinement statistics for the HpDHNA:pterin structure.

Data Collection	
Beamline	22-BM, SER-CAT, APS, ANL
Detector	MARCCD 225
Wavelength (Å)	1.000
Temperature (K)	100
Crystal-to-detector distance (mm)	160
Space group	*I*4
Cell constants	
a, b, c (Å)	68.08, 68.08, 57.40
α, β, γ (°)	90, 90, 90
Resolution (Å)	30–1.49 (1.54–1.49)^a^
Completeness (%)	98.2 (82.6)
Total/Unique reflections	998,205/21,197
Redundancy	14.1/9.4
*I*/σ (*I*)	17.5/1.2
*R*_pim_	0.036 (0.652)
CC_1/2_	0.999 (0.547)
**Refinement**	
Resolution (Å)	26.90–1.49 (1.57–1.49)
No. Of reflections	21,173 (2782)
*R*_work_/*R*_free_	0.199/0.230
No. Of atoms/B-factors (Å^2^)	
Protein	1061/29.17
Ligand	16/33.45
Water	134/41.81
R.m.s. Deviations	
Bond lengths (Å)	0.008
Bond angles (°)	0.990
Ramachandran plot	
Favored (%)	98.23
Allowed (%)	1.77
Outliers (%)	0

a Values in parentheses are for the highest-resolution shell.

### 2.4 Crystal structure solution and refinement

The crystal structure was solved by molecular replacement with PHASER (McCoy et al., 2007) embedded in the PHENIX program suite (Echols et al., 2012). A search model was generated from the crystal structure of EcDHNA in complex with neopterin (NP, Fig. 1B) (Blaszczyk et al., 2014). The sequence identity between HpDHNA and EcDHNA is 22%. The Z scores for rotation and translation functions were 9.3 and 18.8, respectively. The initial model was rebuilt with COOT (Emsley and Cowtan, 2004) and refined with phenix.refine (Afonine et al., 2012) in the PHENIX program suite. The starting *R*_work_ and *R*_free_ values were 0.35 and 0.36, respectively. The refinement at 1.49-Å resolution converged with residuals of *R*_work_ ​= ​0.20 and *R*_free_ ​= ​0.23 with excellent model geometry. Although HP was incubated with HpDHNA for co-crystallization, the initial electron density revealed the existence of pterin in the active site for an unknown reason. The final structure was evaluated by the validation server of Worldwide Protein Data Bank (wwPDB) (Gore et al., 2017). Refinement statistics are summarized in Table 1. Illustrations were prepared using the PyMOL molecular graphics system (Schrödinger, LLC., New York, NY, USA).

### 2.5 Dynamic light scattering (DLS) measurement and analysis

DLS experiment was carried out with the DynaPro NanoStar (Wyatt Technology Corporation, Santa Barbara, CA, USA). Ligand-free HpDHNA sample was prepared in a buffer consisting of 150 ​mM NaCl and 25 ​mM Tris pH 7.5. Three measurements were performed at concentrations 30.4, 10.0, and 1.0 ​mg ​ml^−1^, respectively.

### 2.6 Small-angle X-ray scattering (SAXS) data collection and analysis

The HpDHNA:Pterin sample was prepared in a buffer consisting of 150 ​mM NaCl and 25 ​mM Tris-HCl pH 7.5. Measurements for concentrations 4.0, 2.0, 1.0, and 0.5 ​mg ​ml^−1^ were carried out to remove the scattering contribution due to interparticle interactions and extrapolate the data to infinite dilution during data analysis. The sample solution and matching buffer were measured using a flow cell to minimize radiation damage. The parameters of SAXS data collection are presented in Table 2. The two-dimensional intensity maps were corrected and reduced to one-dimensional scattering profiles using software developed by the 12-ID-B beamline at APS, ANL. The one-dimensional SAXS profiles were averaged after eliminating outliers.

**Table 2:** SAXS data collection and modelling statistics for the HpDHNA:Pterin structure.

Data Collection	
Beamline	12-ID-B, APS, ANL
Detector	PILATUS 2 ​M
Beam geometry (mm)	0.10 ​× ​0.12
Sample flow rate (μL/s)	10
Wavelength (Å)	0.932
Flux (photons/s)	5 ​× ​10^12^
Camera length (m)	2.0
Effective q range (Å^−1^)	0.006–0.880
Total number of sequential data frames	45
Exposure time (s)	1.0
Temperature (K)	298
Sample configuration	Flow cell, effective sample length 1.5 ​mm
**Structural parameters from data extrapolated to infinity dilution**	
Guinier analysis	
*I*(0) (a.u.)	0.0180 ​± ​0.0004
*R*_g_ (Å)	24.7 ​± ​0.7
*P*(r) analysis	
*I*(0) (a.u.) reciprocal space	0.01848
*I*(0) (a.u.) real space	0.0185 ​± ​0.0003
*R*_g_ (Å) reciprocal space	24.6
*R*_g_ (Å) real space	24.6 ​± ​0.4
*D*_max_ (Å)	72.9
Porod volume (Å^3^)	76,500
χ^2^ (total estimate from GNOM)	0.8860
Molecular weight (MW) determination	
MW based on V_app_ (kDa)	53.4
MW based on V_c_ (kDa)	55.3
Oligomer status	4
**Modelling**	
Computation of model intensities (CRYSOL)	Tetrameric HpDHNA:Pterin (this work)
χ^2^	0.086
*R*_g_ from slope of net intensity (Å)	24.65
Molecular weight (kDa)	54.3
Vol (Å^3^), Ra (Å), Dro (e Å^−3^)	72,556, 1.740, 0.035
Ab initio model by DAMMIN	
Average excluded volume (Å^3^)	75658.5
NSD from 32 runs	0.75 ​± ​0.05

Buffer background subtraction and intensity extrapolation to infinite dilution were carried out using the MatLab script developed by the 12-ID-B beamline at APS, ANL. The radius of gyration (*R*_g_) was generated from Guinier plot of the data extrapolated to infinite dilution in the range of *qR*_g_ ​< ​1.3. For comparison, *R*_g_ was also calculated in real space and reciprocal space using program GNOM in q range up to 0.30 ​Å^−1^ (Svergun, 1992). Pair-distance distribution function *P*(r) and maximum dimension (*D*_max_) were also calculated using GNOM. Theoretical solution scattering intensity and *R*_g_ from crystal structure were calculated and fitted to the experimental scattering intensity using CRYSOL (Svergun et al., 1995). Molecular weight (MW) was estimated using two methods based respectively on apparent volume, V_app_ (Fisher et al., 2010), and correlation volume, V_c_ (Rambo and Tainer, 2013) (Table 2). Thirty-two ab initio shape reconstructions (molecular envelopes) were generated independently using DAMMIN in slow mode and then averaged and filtered (Volkov and Svergun, 2003).

## 3 Results and discussion

### 3.1 Tetrameric form of the HpDHNA:Pterin structure

The asymmetric unit of the HpDHNA:Pterin structure contains one GGG-DHNA polypeptide, one pterin molecule, one ethylene glycol molecule, and 134 water molecules. The first two glycine residues at the N terminus (G-2 and G-1) and three residues at the C terminus (N115, N116, and L117) were not observed and presumably disordered.

The M1-E114 sequence of the HpDHNA protein folds into a four-stranded antiparallel β-sheet (β1, β2, β3, and β4) flanked with three α-helices (α1, α3, and α4) (Fig. 1C). Unlike other DHNAs, the α2 and 3_10_ helices are not observed in HpDHNA apparently due to a six-residue sequence deletion (Fig. 1D). Like other DHNAs, the active site of HpDHNA is formed by catalytic residues from two adjacent subunits in a tetramer. Unlike other DHNAs, however, the HpDHNA tetramer does not form a head-to-head octamer. Interestingly, MtDHNA in complex with HP (MtDHNA:HP) forms an octamer, but the apo-MtDHNA structure forms a tetramer (Fig. 1D), which is the only tetrameric DHNA structure published to date.

**Fig. 2:**
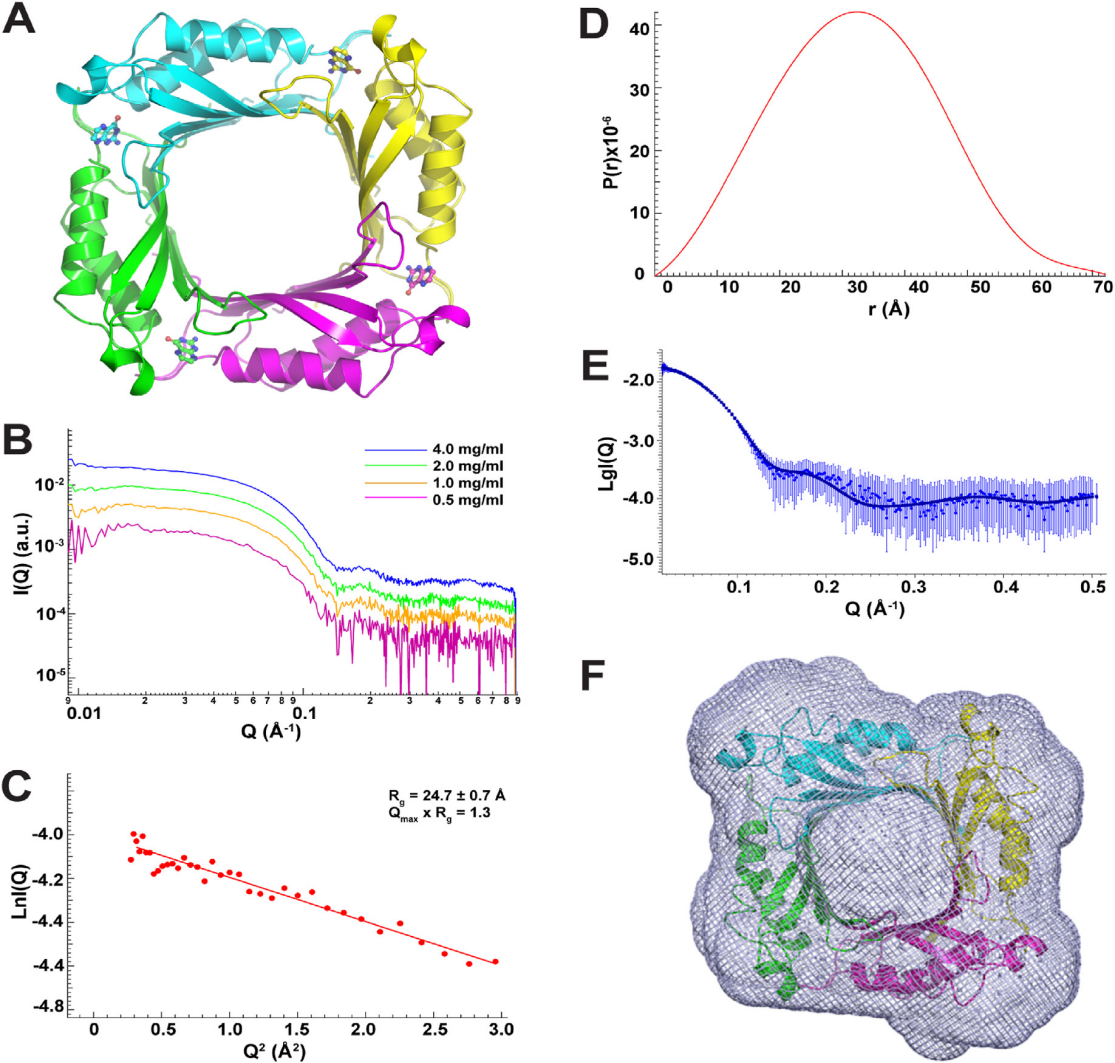
Tetrameric HpDHNA:Pterin complex in crystal and in solution. (**A**) Crystal structure of the HpDHNA:Pterin tetramer colored by subunits. (**B**) Measured SAXS profile I vs Q for the complex as function of concentration. (**C**) The Guinier plot of low-angle portion of the scattering data is linear, consistent with a monodisperse solution. (**D**) The pair distribution function P(r) for the complex. (**E**) Fit of the crystal structure of the complex to the experimental date. The dots are experimental data, and the solid line is the solution scattering profile calculated from the crystal structure. (**F**) Overlay of the SAXS-derived ab initio shape envelope with the tetrameric crystal structure.

The HpDHNA:Pterin complex exists as a tetramer in the crystal lattice. The hydrophobic core of HpDHNA is formed between the β-sheet and helices α3 and α4. The HpDHNA:Pterin complex forms a donut-shaped tetramer with four identical subunits related by a four-fold axis (Fig. 2A). In the tetramer, β1 of each subunit reaches β4 of neighboring subunit with 11 hydrogen bonds, resulting in a sixteen-stranded antiparallel β-sheet in the shape of a perfect barrel wall (Fig. 2A). In addition, each β1-β4 interaction is enforced by two salt bridges. The outer diameter, inner diameter, and height of the tetramer are approximately 71, 13, and 45 ​Å, respectively.

### 3.2 The tetrameric HpDHNA:Pterin complex is also observed in solution

To investigate the oligomeric form of ligand-free HpDHNA in solution, we performed DLS measurements at three concentrations, starting from 30.4 ​mg ​ml^−1^ and ending at 1.0 ​mg ​ml^−1^, showing that apo-HpDHNA exists as a tetramer in solution (Table 3). To elucidate the oligomeric form of the HpDHNA:Pterin complex in solution, we collected SAXS data at four concentrations, starting from 4.0 ​mg ​ml^−1^, slightly higher than that for crystallization (3.7 ​mg ​ml^−1^), and ending at 0.5 ​mg ​ml^−1^. Fig. 2B shows the SAXS scattering profiles from the HpDHNA:Pterin complex as the function of concentration. The data extrapolated to zero concentration (infinite dilution) were used for further analysis, including estimation of MW, generation of structural parameters, and fitting/modelling. The estimated MW is ∼55 ​kDa (Table 2), confirming that the HpDHNA:Pterin complex exists as a tetramer in solution (MW of the monomer is 13.8 ​kDa). Linearity of the Guinier plot indicates that the HpDHNA:Pterin complex does not aggregate in solution (Fig. 2C). The *R*_g_ determined from the Guinier plot agrees well with the *R*_g_ determined from the *P*(r) analysis (Table 2). The *D*_max_, determined from the *P*(r) function (Fig. 2D), is 72.9 ​Å (Table 2). The symmetrical, bell-shaped *P*(r) distribution indicates that the HpDHNA:Pterin complex forms globular, compact particles. The solution scattering profile calculated from the crystal structure fits well to the experimental scattering curves from SAXS with a χ^2^ value of 0.886 (Fig. 2E). Also, the *R*_g_ (24.65 ​Å) and *D*_max_ (72.32 ​Å) values calculated from the crystal structure agree with the SAXS experimental results (Table 2). The SAXS-derived molecular envelope, obtained based on 32 independent DAMMIN runs in four-fold symmetry, fits well with the crystal structure of the HpDHNA:Pterin tetramer (Fig. 2F).

**Table 3:** Dynamic light scattering (DLS) measurements for ligand-free HpDHNA.

Protein Concentration (mg ml^−1^)	Molecular Weight (kDa)	Oligomeric State
30.4	61.0 ​± ​5.7	tetramer
10.0	61.0 ​± ​5.7	tetramer
1.0	66.0 ​± ​12.1	tetramer

### 3.3 Tetrameric HpDHNA:Pterin complex exhibits well-formed active sites

**Fig. 3:**
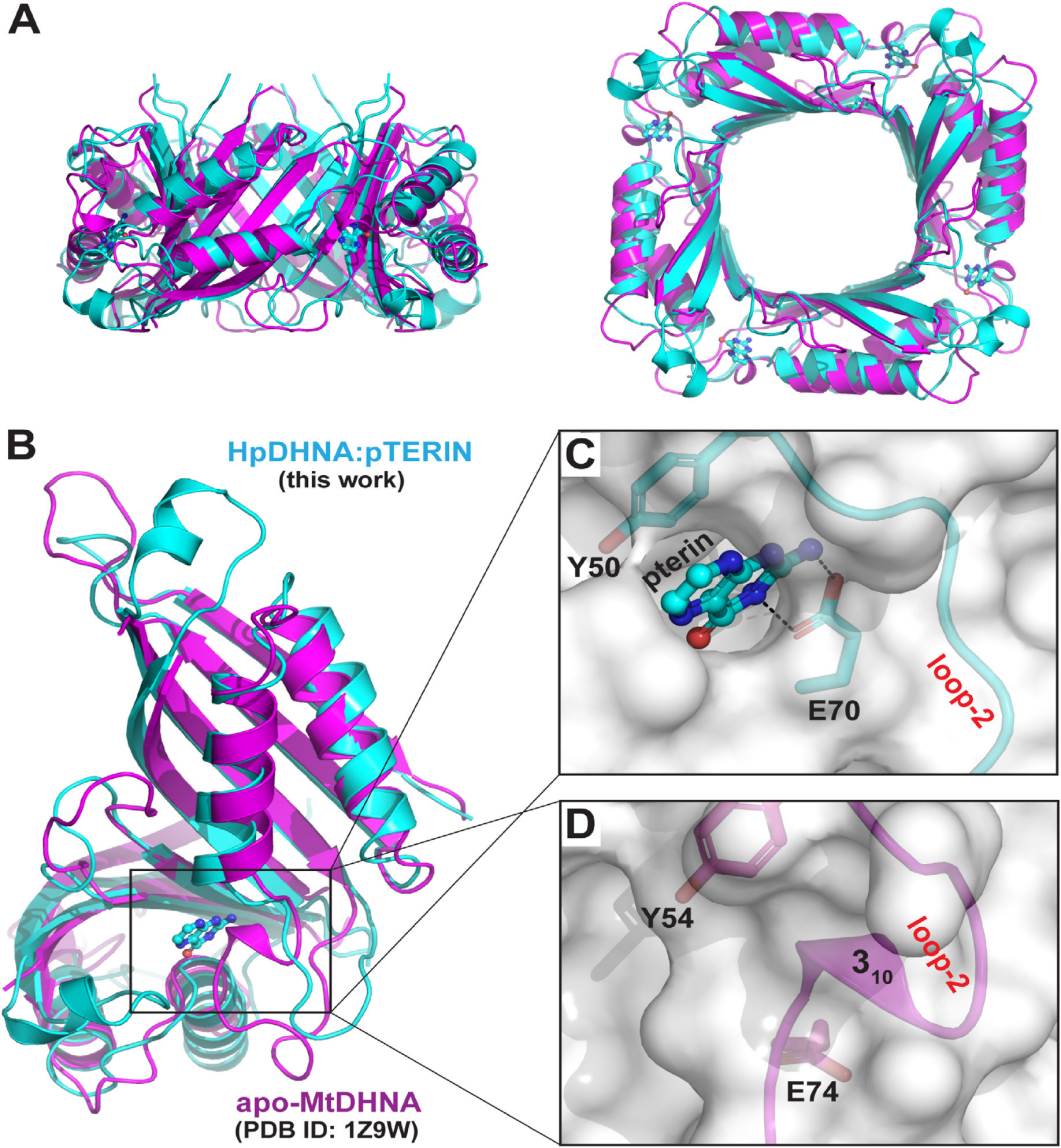
Active site architecture of HpDHNA:Pterin and apo-MtDHNA. (**A**) Two views of superimposed structures of HpDHNA:Pterin (in cyan, this work) and apo-MtDHNA (in magenta, PDB ID: 1Z9W). Proteins are shown as ribbon diagrams (helices as spirals, strands as arrows, and loops as tubes) and pterin as a ball-and-stick model in atomic color scheme (N, blue; C, cyan; and O, red). (**B**) Two subunits of tetrameric HpDHNA:Pterin and apo-MtDHNA structures are superimposed. (**C, D**) Zoomed-in view around the active site of the two structures outlined with transparent molecular surfaces. Strictly conserved Glu and Tyr side chains are shown as sticks. Hydrogen bonds are indicated with dashed lines. (For interpretation of the references to color in this figure legend, the reader is referred to the Web version of this article.)

DHNA structures share four flexible regions, namely loop-1, loop-2, loop-3, and loop-4 (Fig. 1D). Loop-1 features an embedded α-helix (α1). Loop-2 contains not only an α-helix (α2), but also a 3_10_-helix (3_10_). Whereas the α1, α2, and 3_10_ are observed in octameric DHNA structures, not all of them are observed in tetrameric structures of apo-MtDHNA (Goulding et al., 2005) and HpDHNA:Pterin. In apo-MtDHNA, α1 is not observed because the entire loop-1 is disordered; in addition, α2 unfolds within loop-2 although the 3_10_-helix is formed. In HpDHNA:Pterin, a six-residue deletion eliminates not only α2, but also the formation of 3_10_ (Fig. 1D). Nonetheless, these uncommon features in the loop-1 and loop-2 regions do not change the overall DHNA fold and the formation of tetrameric apo-MtDHNA and HpDHNA:Pterin complexes. As shown, the two tetramers are superimposed well (Fig. 3A). The root-mean-square deviation for 308 out of 345 pairs of aligned Cα positions is 1.48 ​Å. However, the structural changes in loop-1 and loop-2 in apo-MtDHNA and HpDHNA:Pterin have significant impact on the formation of their active sites.

Since the active site of DHNA is located between two subunits, a DHNA tetramer has four identical active sites (Fig. 3A, right panel). To compare one of the four active sites, we need to superimpose two subunits of apo-MtDHNA and HpDHNA:Pterin structures (Fig. 3B). As shown, the 3_10_-helix of apo-MtDHNA and the pterin molecule in HpDHNA:Pterin are in proximity of each other. As such, the ligand-binding site seen in HpDHNA:Pterin (Fig. 3C) is partially blocked by the 3_10_-helix in apo-MtDHNA (Fig. 3D). Furthermore, the E74 side chain for ligand recognition (Fig. 3C) is pointing into an opposite direction (Fig. 3D). It appears that the unfolding of α2 in apo-MtDHNA relocates the 3_10_-helix and the shifted 3_10_ blocks part of the active site (Fig. 3D), whereas the deletion of α2 in HpDHNA:Pterin unfolds the 3_10_-helix but the resulting loop is distant from well-formed active site (Fig. 3C).

**Fig. 4:**
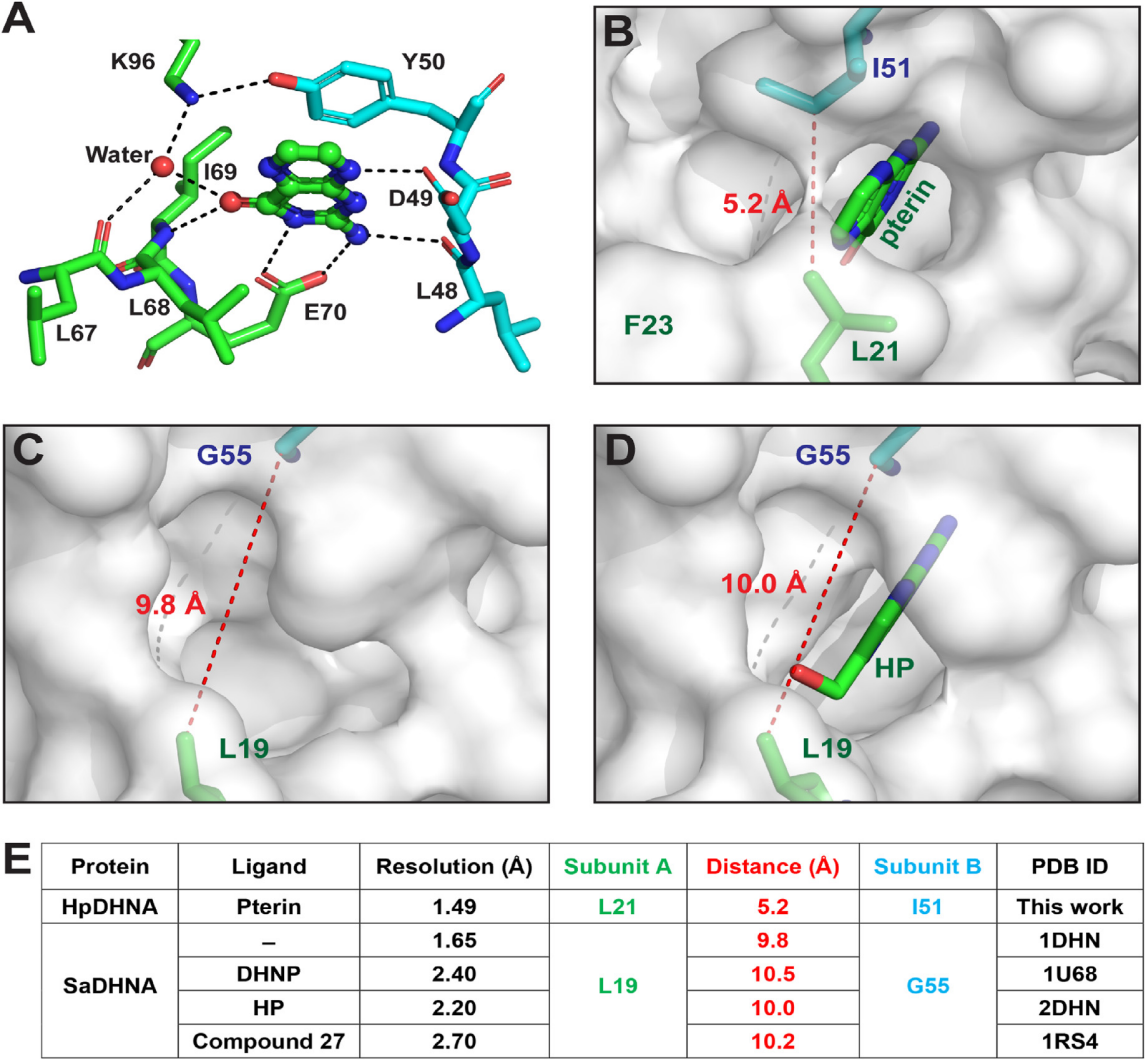
Active site architecture in HpDHNA and SaDHNA. (**A**) HpDHNA recognizes pterin via hydrogen bonding and π-π stacking. Amino acid residues are illustrated as stick models and pterin as a ball-and-stick model in atomic color scheme (N in blue, C in green or cyan, and O in red). Hydrogen bonds are indicated with dashed lines. (**B**) The contact distance between L21 and I51 in HpDHNA:pterin (this work) is indicated with a dashed line in red. The protein is outlined with a transparent surface. (**C**, **D**) The contact distances between L19 and G55 in SaDHNA are indicated for apo-SaDHNA (PDB ID: 1DHN) and SaDHNA:HP (PDB ID: 2DHN). (**E**) The L19/G55 contact distance in SaDHNA, either ligand free, or in complex with substrate DHNP, product HP, or inhibitor compound **27**, is a characteristic feature for a wide-open active site. (For interpretation of the references to color in this figure legend, the reader is referred to the Web version of this article.)

The pterin molecule is buried deep in the active site of HpDHNA (Fig. 3C). It is recognized by the enzyme via π-π stacking with residue Y50 and hydrogen bonding with residues D49, L48, E70, and I69 (Fig. 4A). In addition, the pterin molecule also forms a hydrogen bond with a water molecule that is held in place by hydrogen bonding with residues L67 and K96 (Fig. 4A). Among above-mentioned residues, Y50, E70, and K96 are strictly conserved among DHNA sequences (Fig. 1D); furthermore, the bridging water molecule is also observed in other DHNA structures, such as SaDHNA:NP (Blaszczyk et al., 2007) and EcDHNA:NP complexes (Blaszczyk et al., 2014). Whereas Y50 and E70 are essential for substrate recognition, K96 and the conserved water molecule are required for DHNA catalysis (Blaszczyk et al., 2014; Wang et al., 2007). Therefore, the active site in the tetrameric HpDHNA:Pterin complex is well formed, providing structural basis for catalytic activity.

### 3.4 An isozyme-specific structural feature of DHNA active sites

**Fig. 5:**
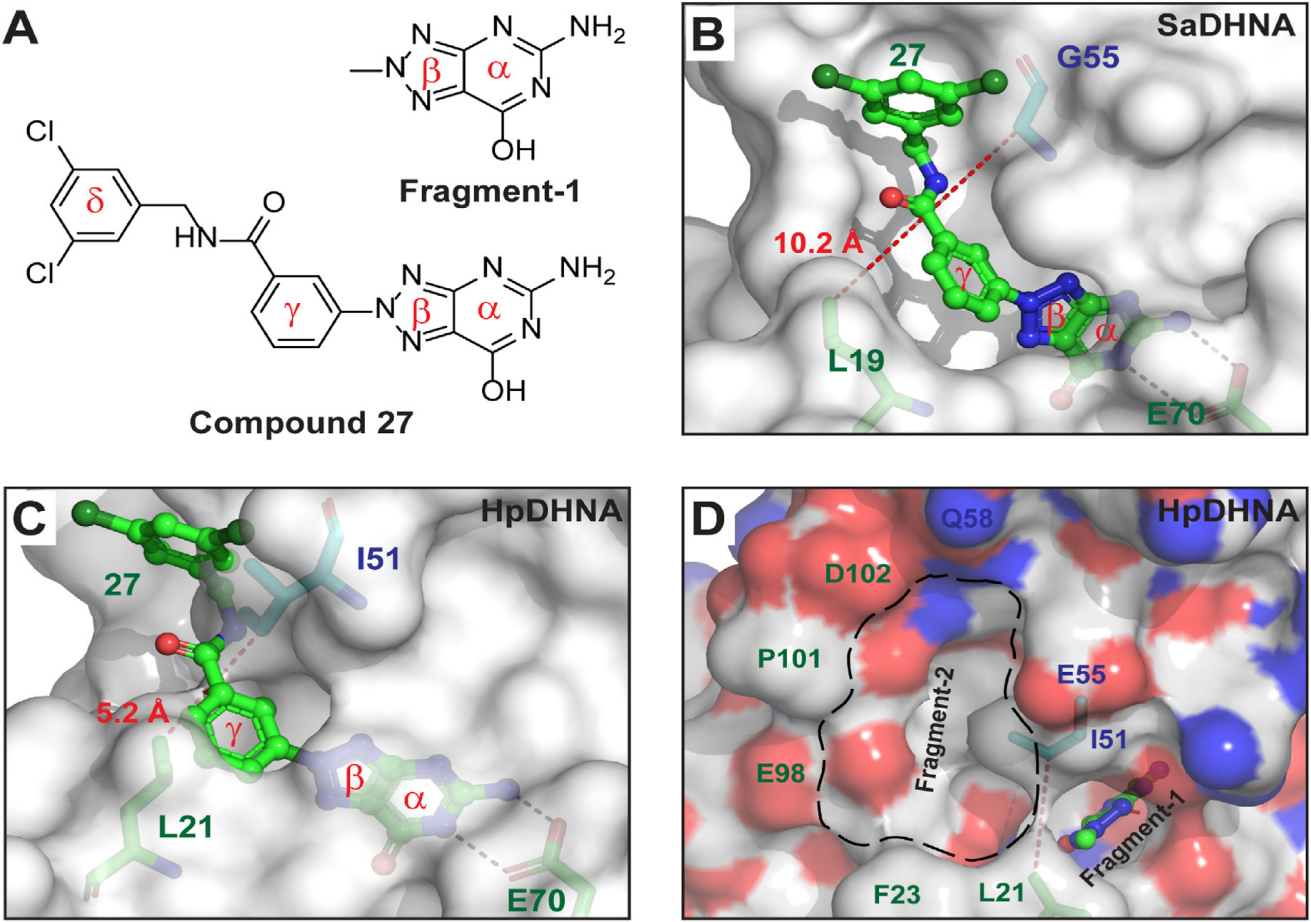
A strategy for fragment-based design of HpDHNA-specific inhibitors. (**A**) Structure of compound **27**, of which the four ring systems are labeled. (**B**) As revealed by the SaDHNA:Compound **27** structure (PDB ID: 1RS4), the wide-open active site with an extended binding groove accommodates the inhibitor (ball-and-stick model: N in blue, C in green, O in red, and Cl in dark green). (**C**) In contrast, the active site of HpDHNA is blocked by side chains L21 and I51 that conflict with ring-γ and ring-δ of compound **27**, respectively. (**D**) Fragment-based approach toward HpDHNA-specific inhibitors appears to be feasible with the fused ring system of compound **27** as Fragment-1, close to which a potential binding site for Fragment-2 is outlined with a dashed line in black on a transparent molecular surface in atomic color scheme (N in blue, C in white, and O in red). (For interpretation of the references to color in this figure legend, the reader is referred to the Web version of this article.)

Although pterin recognition by HpDHNA (Fig. 4A) exhibits common features of ligand binding by DHNAs, the exit of HpDHNA's active site is blocked by residues L21 and I51 (Fig. 4B). The contact distance between L21 and I51 is only 5.2 ​Å. The counterpart of HpDHNA's L21/I51 in SaDHNA is L19/G55 (Fig. 1D). The contact distances between L19 and G55 are 9.8 and 10.0 ​Å in apo-SaDHNA (Fig. 4C) and SaDHNA:HP (Fig. 4D), respectively, characterizing a wide-open active site. Furthermore, this contact distance remains almost constant in apo-SaDHNA, SaDHNA:DHNP (substrate complex), SaDHNA:HP (product complex), and SaDHNA:Compound-**27** (inhibitor complex) (Fig. 4E). The variation of this contact distance is negligible despite the fact that the active site exit does not look the same in different states as shown for apo-SaDHNA (Fig. 4C) and SaDHNA:HP (Fig. 4D). These observations demonstrate that the SaDHNA-specific contact distance of ∼10 ​Å is a characteristic feature of its active site. Although compound **27** (inhibitor, Fig. 5A) is significantly larger than DHNP and HP (Fig. 1A), it does not change the isozyme-specific contact distance (ISCD). Previously, A blocked active site has also been observed for *Yesinia pestis* DHNA (YpDHNA). The ISCD values in apo-YpDHNA (PDB ID: 3R2E) and YpDHNA:Pterin (PDB ID: 6OJO) are 6.3 ​Å and 6.4 ​Å, respectively. We propose that the ISCD is a characteristic feature of DHNA active sites, wide-open or blocked. It will be confirmed when structures of each isozyme in multiple forms (ligand-free, substrate complex, product complex, etc.) become available.

The ISCD not only characterizes DHNA active sites, but also provides structural basis for distinct enzymatic activity of DHNA isozymes. Previously, we reported a 1.07-Å structure of EcDHNA in complex with NP (Blaszczyk et al., 2014). Reviewing this structure, we found that EcDHNA also has a blocked active site and that the ISCD value of a blocked active site can be as small as ∼4 ​Å. Remarkably, earlier results from NMR, equilibrium binding, and transient kinetic analyses show that EcDHNA and SaDHNA have significantly different binding and catalytic properties (Wang et al., 2007). For example, the binding affinity of HP for EcDHNA and SaDHNA are 0.4 and 24.0 ​μM, respectively, showing that a blocked active site binds HP much tighter than a wide-open active site. Like EcDHNA, HpDHNA also has a blocked active site; therefore, it must also bind HP much tighter than SaDHNA.

### 3.5 A fragment-based strategy for HpDHNA-specific inhibitor design

As mentioned above, compound **27**, with an IC_50_ value of 68 ​nM, is the most potent among a family of SaDHNA inhibitors (Sanders et al., 2004). The compound contains ring systems α, β, γ, and δ, among which ring-α and ring-β are fused (Fig. 5A). The active site of SaDHNA features a wide-open exit with an extended binding groove. Whereas the fused α and β rings of compound **27** fit well in the active site, the γ and δ moieties are accommodated by the extended binding groove (Fig. 5B). Unlike SaDHNA, HpDHNA has a blocked active site, and the nearby landscape is also different from SaDHNA (Fig. 5C). To find out whether compound **27** could bind to HpDHNA, we superimposed the SaDHNA:Compound-**27** and HpDHNA:Pterin structures and simply placed compound **27** in the active site of HpDHNA, showing that the collisions between L21/I51 and ring-γ/ring-δ are unavoidable (Fig. 5C). Although compound **27** may not bind to HpDHNA, its ring-α/ring-β moiety fits well in the active site of HpDHNA, suggesting a fragment-based strategy for the development of HpDHNA-specific inhibitors.

Fragment-based drug discovery (FBDD), conceptualized 41 years ago (Jencks, 1981), has emerged as a highly successful approach to identify quality leads for optimization into approved medicines and clinical candidates (Erlanson et al., 2016), including four drugs on the market and more than 40 new chemical entities in clinical trials (Konteatis, 2021). Starting with fragment screening, a typical FBDD procedure involves fragment linking, merging, and growing approaches during hit-to-lead and lead-optimization stages. Toward HpDHNA, compound **27** has already provided the fused ring-α and ring-β as an excellent fragment (Fragment-1, Fig. 5A). It has two important features. First, Fragment-1 is not a pterin-like structure, and therefore, may not have a cell permeability problem due to the lack of a folate transporter in microorganisms. Second, Fragment-1 can be readily recognized by HpDHNA with conserved structural features inside the active site of DHNAs, especially E70 (Fig. 5C). Nonetheless, Fragment-1 does not provide isozyme specificity. Additional fragments are needed to achieve HpDHNA specificity, for which a nearby binding pocket is readily available (Fig. 5D). This potential binding site features a hydrophobic bottom, backbone amide/carbonyl groups along the wall, and sidechain amino/carboxyl groups along the edge. Assuming an additional fragment (Fragment-2) could be identified for this binding site, a linker could be built to reach Fragment-1 either under or above the L21-I51 ISCD bridge (Fig. 5D), creating a potential inhibitor specifically targeting HpDHNA. Both fragment soaking and virtual screening are feasible approaches. This strategy may also be feasible for inhibitor design targeting other DHNAs, depending upon whether a potential binding site for Fragment-2 is available near the binding site of Fragment-1.

## 4 Conclusions

Folate cofactors are essential for life. Mammals obtain folates from their diets, whereas microorganisms must synthesize folates *de novo*. Therefore, enzymes in folate biosynthesis pathway are potential targets of antimicrobial agents. The beginning of modern antimicrobial chemotherapy is represented by the clinical use of Sulfonamides. These sulfa drugs target DHPS, one of the four mid-pathway enzymes (DHNA, HPPK, DHPS, and DHFS) that do not have counterparts in mammals. *H. pylori* is a widespread bacterial pathogen, growing in human stomach of over 50% of the world population. It is responsible for considerable health risks including the development of gastric ulcers, but the treatment of *H. pylori* infection is difficult because of its high resistance to antibiotics. Joining the fight against *H. pylori*, we have determined the crystal structure of *H. pylori* (strain G27) DHNA in complex with pterin. The structure represents the first tetrameric DHNA complex with well-formed active sites. The active site is, however, blocked by two amino acid residues at the exit, between which the contact distance is 5.2 ​Å. Based on a wealth of structural data, we find that this contact distance is independent of ligand binding and that it is isozyme specific. For example, it is ∼10 ​Å for SaDHNA as observed in the crystal structures of apo-SaDHNA, SaDHNA:DHNP, SaDHNA:HP, and SaDHNA:compound **27**, whereas it is ∼6.5 ​Å for *Yesinia pestis* DHNA (YpDHNA) as observed in apo-YpDHNA (PDB ID: 3R2E) and YpDHNA:Pterin (PDB ID: 6OJO). Accordingly, we name it isozyme-specific contact distance and propose that ISCD is a characteristic structural feature of all DHNA isozymes. Our hypothesis will be confirmed when more structures of DHNA isozymes in both ligand-free and ligand-bound states become available. Our comparative analysis of DHNA structures also suggests a fragment-based strategy for HpDHNA-specific inhibitor design, which may also be applicable to inhibitor design targeting other isozymes.

## Accession codes

Atomic coordinates and structure factors have been deposited in the Protein Data Bank (Burley et al., 2017) with accession code 8EVK.

## Funding information

This research was supported by the Intramural Research Program of the 10.13039/100000002National Institutes of Health, 10.13039/100000054National Cancer Institute, Center for Cancer Research.

## CRediT authorship contribution statement

**Gary X. Shaw:** Data curation, Formal analysis, Visualization, Writing – original draft. **Lixin Fan:** Data curation, Formal analysis, Visualization, Writing – original draft. **Scott Cherry:** Resources, Writing – original draft. **Genbin Shi:** Resources. **Joseph E. Tropea:** Resources, Supervision, Writing – original draft. **Xinhua Ji:** Conceptualization, Project administration, Supervision, Validation, Writing – review & editing.

## Declaration of competing interest

The authors declare that they have no known competing financial interests or personal relationships that could have appeared to influence the work reported in this paper.

## Data Availability

Atomic coordinates and structure factors have been deposited in the Protein Data Bank (PDB) with accession code 8EVK.
